# Delayed Pathogenicity in *Toxoplasma gondii* as an Evolutionary Framework for Late-Life Disease and Mortality

**DOI:** 10.3390/microorganisms14081757

**Published:** 2026-08-10

**Authors:** Ariel Israel, Sarah Israel

**Affiliations:** 1 Leumit Research Institute, Leumit Health Services, 23 Sprinzak St., Tel Aviv 6473817, Israel; 2 Department of Epidemiology and Preventive Medicine, School of Public Health, Gray Faculty of Medical & Health Sciences, Tel Aviv University, Tel Aviv 6997801, Israel; 3 Faculty of Medicine, Hebrew University of Jerusalem, Jerusalem 9112001, Israel; 4 Department of Clinical Microbiology and Infectious Diseases, Hadassah Medical Center, Jerusalem 9112001, Israel

**Keywords:** *Toxoplasma gondii*, delayed pathogenicity, latent infection, bradyzoites, evolutionary ecology, aging and chronic disease, neurodegeneration, colorectal cancer, felid gut

## Abstract

*Toxoplasma gondii* infects most warm-blooded vertebrates and establishes lifelong persistence through latent bradyzoite cysts within long-lived tissues. We propose that this persistence may represent an evolved strategy of delayed pathogenicity. Because *T. gondii* completes sexual reproduction only in felids, most productively through ingestion of infected tissues, transmission may be favored by host impairments that increase vulnerability to predation or scavenging. However, pathogenic effects expressed too early could compromise host reproduction and reservoir maintenance. We therefore hypothesize that evolutionary pressures may favor pathogenic effects emerging predominantly later in host life, after reproduction has largely been achieved. Pharmacologic protozoal suppression has been associated with reduced mortality and multiple chronic diseases, while *T. gondii* DNA increases along the adenoma–carcinoma sequence in gastrointestinal malignancies. Together, these observations motivate a testable framework linking long-term parasite persistence to late-life morbidity.

## 1. Introduction

*Toxoplasma gondii* is a protozoan parasite that establishes lifelong infection in most warm-blooded vertebrates [1,2]. Yet its sexual reproduction occurs exclusively in felids and depends on ingestion of infected tissues, a process ultimately incompatible with survival of the intermediate (prey) host. This life cycle imposes an unusual evolutionary constraint. Predation or scavenging of infected hosts is required for parasite amplification, but excessive pathogenicity expressed before host maturation and reproduction could compromise the ecological reservoirs on which transmission depends. We therefore propose that bradyzoite persistence represents an evolved strategy of delayed pathogenicity. Under this framework, pathogenic effects that increase vulnerability to predation or scavenging are preferentially expressed later in life, after reproductive success has largely been achieved and with reduced impact on host population continuity.

Felids can acquire *T. gondii* by routes other than carnivory, by ingesting sporozoites in oocysts or tachyzoites, and, less commonly, congenitally. These routes are not equivalent as sources of amplification. Near-universal oocyst shedding follows the ingestion of bradyzoite-laden tissue, with a prepatent period of 3–10 days, whereas fewer than half of felids shed after ingesting oocysts or tachyzoites, and then only after 18 days or more [3,4,5]. The difference is not only one of frequency but of the stage produced, and it is the stage that determines how far the parasite can travel. A felid that has ingested bradyzoite-infected prey may shed as many as several hundred million oocysts over one to two weeks [6], and sporulated oocysts remain infective outside any host for extraordinary periods—18 months in soil, and 24 months in seawater held at 4 °C [7,8]. The carnivorous route is therefore the only one that converts a single transmission event into a durable and spatially distributed source of infection for a large number of subsequent hosts; the alternative routes yield either no amplification or a greatly diminished one. The argument advanced here rests on this asymmetry in amplification, not on the exclusivity of infection: ingestion of infected tissue is not the only route by which a felid becomes infected, but it is by a wide margin the most productive route to the oocyst.

## 2. A Unique Evolutionary Constraint: Host Death Is Required for Pathogen Amplification

Most pathogens gain little by harming their hosts: their success depends on replication and transmission between living hosts, and excessive virulence is usually selected against [9,10,11,12]. *T. gondii* belongs to a distinct ecological class. Its sexual reproduction occurs exclusively in the intestines of felids that consume infected host tissues [2,13], a step essential for genetic recombination and for producing resistant oocysts in the massive numbers that enable large-scale environmental spread [14]. Predation or scavenging, which typically involve host death, is a crucial step in the parasite’s life cycle and therefore impose strong selection for traits that facilitate felid consumption of infected hosts [15]. Loss of aversion to cat odor provides one mechanism by which this process occurs in rodents [16,17]. However, *T. gondii*’s capacity to spread through a wide variety of host species, including birds, livestock, and primates [18,19,20], is not explained by rodent-specific behavioral interactions. Any physiological, neurological, or systemic impairment that increases vulnerability to predation or scavenging can serve the same reproductive function across host species.

At the same time, pathogenic effects expressed early in life may reduce opportunities for host growth, maturation, and reproduction. More importantly, excessive mortality before completion of a reproductive window could diminish the ecological reservoirs on which transmission depends. These constraints would be expected to create selective pressure favoring parasite traits that increase susceptibility to feline predation while preserving host maturation and reproductive success. Under this framework, pathogenic effects facilitating transmission would be predicted to emerge preferentially after reproduction has largely been achieved, because parasite strategies that substantially impair host reproductive success may ultimately reduce long-term transmission opportunities.

Moreover, in species in which reproductive potential declines with age, host impairments that emerge after reproduction fall into a period when natural selection on the host is reduced [21,22]. Defenses against late-onset pathology confer little reproductive advantage and thus would spread poorly. By concentrating pathogenic effects in this post-reproductive “selection shadow,” a parasite may maximize transmission while minimizing host counter-adaptation.

Three qualifications follow. The first concerns scope, because *T. gondii* has no single host. The framework applies to intermediate hosts that (i) are subject to felid predation or scavenging and (ii) have reproductive schedules such that a non-negligible fraction of individuals survive beyond peak reproductive output. Felids span a wide size range, and their prey accordingly ranges from rodents and birds to ungulates as large as buffalo; the argument is not restricted to small-bodied hosts, nor does it require that infection be acquired early in life. What is proposed to be deferred is the pathogenic transition, not the establishment of infection, and a program that withholds impairment until host reproductive value has declined operates whenever infection is acquired. Humans need not satisfy these conditions at all. Selection need not have acted in humans for humans to experience the consequences of the parasite’s reproductive cycle. We are incidental, effectively dead-end hosts, and the claim advanced here is therefore not that our late-life pathology is adaptive for the parasite, but that it is the spillover of a developmental program shaped in prey species, expressed in a host with a long post-reproductive lifespan.

The second concerns acute infection, which in immunocompetent intermediate hosts is characteristically mild and rarely fatal despite rapid tachyzoite proliferation. Two explanations are available, and they are not mutually exclusive. The conventional virulence–transmission trade-off predicts low acute virulence because tissue-cyst transmission requires the host to survive long enough to be eaten. The framework advanced here adds a temporal component: that pathogenic expression is not merely suppressed but deferred, and deferred to a stage of host life at which host death is least costly to the reservoir. The observation of benign acute infection is consistent with both accounts and does not distinguish them. Section 6 sets out what would.

The third concerns parasite genetic diversity. The mechanism proposed here is a general one, following from the reproductive cycle of the parasite rather than from the properties of any particular lineage. It is notable in this connection that the same rhoptry effectors that govern strain-dependent virulence, ROP18 and ROP5, are the effectors that defeat host GTPase loading onto the parasitophorous vacuole (Section 4.2): strain differences in acute virulence and the parasite’s engagement with host cell-autonomous immunity are two faces of one molecular system [23,24]. In North America and Europe, the population structure of *T. gondii* is dominated by a small number of clonal lineages [25,26,27]. South America harbors extensive non-archetypal diversity, and atypical strains there have been associated with severe, occasionally fatal acute disease in immunocompetent people [28]. Asymptomatic or benign acute infection remains the rule in immunocompetent hosts wherever it has been examined, including in the settings where these strains circulate [29]; the severe cases are rare, 44 over a nine-year period in French Guiana, and because atypical strains have seldom been genotyped from asymptomatic infections there, the proportion of such infections that becomes severe is unknown.

## 3. Strategic Latency Through Bradyzoite Persistence

When infecting a host, *T. gondii* transitions from rapidly replicating tachyzoites to encysted bradyzoites. These cysts persist for decades in long-lived tissues such as neurons and skeletal muscle under immune surveillance [30]. Antigen expression is reduced, and the infection is termed “latent,” a state commonly regarded as innocuous and clinically silent in immunocompetent humans [13].

However, bradyzoite persistence is not a passive state. The parasite selectively encysts within long-lived tissues, evades immune elimination, and maintains complex interactions with host regulatory pathways. Recent work further demonstrates substantial developmental heterogeneity among bradyzoites, revealing multiple transcriptionally distinct subpopulations and indicating that the cyst stage represents an active developmental state rather than a uniform dormant endpoint [31]. These observations imply that persistence is under active evolutionary maintenance. While such adaptations may serve multiple functions, they are consistent with a transmission-oriented role in which long-term tissue persistence allows pathogenic effects to be expressed at stages of host life when their potential contribution to parasite transmission is greatest.

Instead, latency may be understood as an adaptive strategy that allows the parasite to persist in host tissues until the host has reached a stage at which mortality would no longer substantially reduce the pool of reproductively capable hosts necessary to ensure local persistence of the host population. In species with age-limited reproductive potential, this stage typically overlaps the post-reproductive period (e.g., menopause or andropause). At that stage, predation may become advantageous for parasite transmission, and host debilitation may increase the probability that infected tissues reach a felid without compromising transmission dynamics. From an evolutionary perspective, latency is therefore unlikely to represent a benign stalemate, but rather an adapted transmission strategy that defers pathogenic effects to a stage of host life when such damage can occur without threatening the parasite’s reservoir.

## 4. Detecting Delayed Pathogenicity

Traditional infectious disease paradigms, historically influenced by Koch’s postulates and their emphasis on consistent proximate outcomes from clearly identifiable infectious events, are not well-suited to detect delayed, heterogeneous, and host-dependent effects of a ubiquitous pathogen capable of long-term stealth persistence within localized tissue niches. Recently, large-scale longitudinal electronic health records have made it possible to observe, over long time scales and without recall bias, what happens when an extremely prevalent persistent pathogen is incidentally suppressed. The resulting epidemiologic signals are strong and difficult to reconcile with the notion that latent toxoplasmosis is biologically inert [32].

### 4.1. Protozoal Suppression, Mortality and Late-Life Morbidity

**Figure 1:**
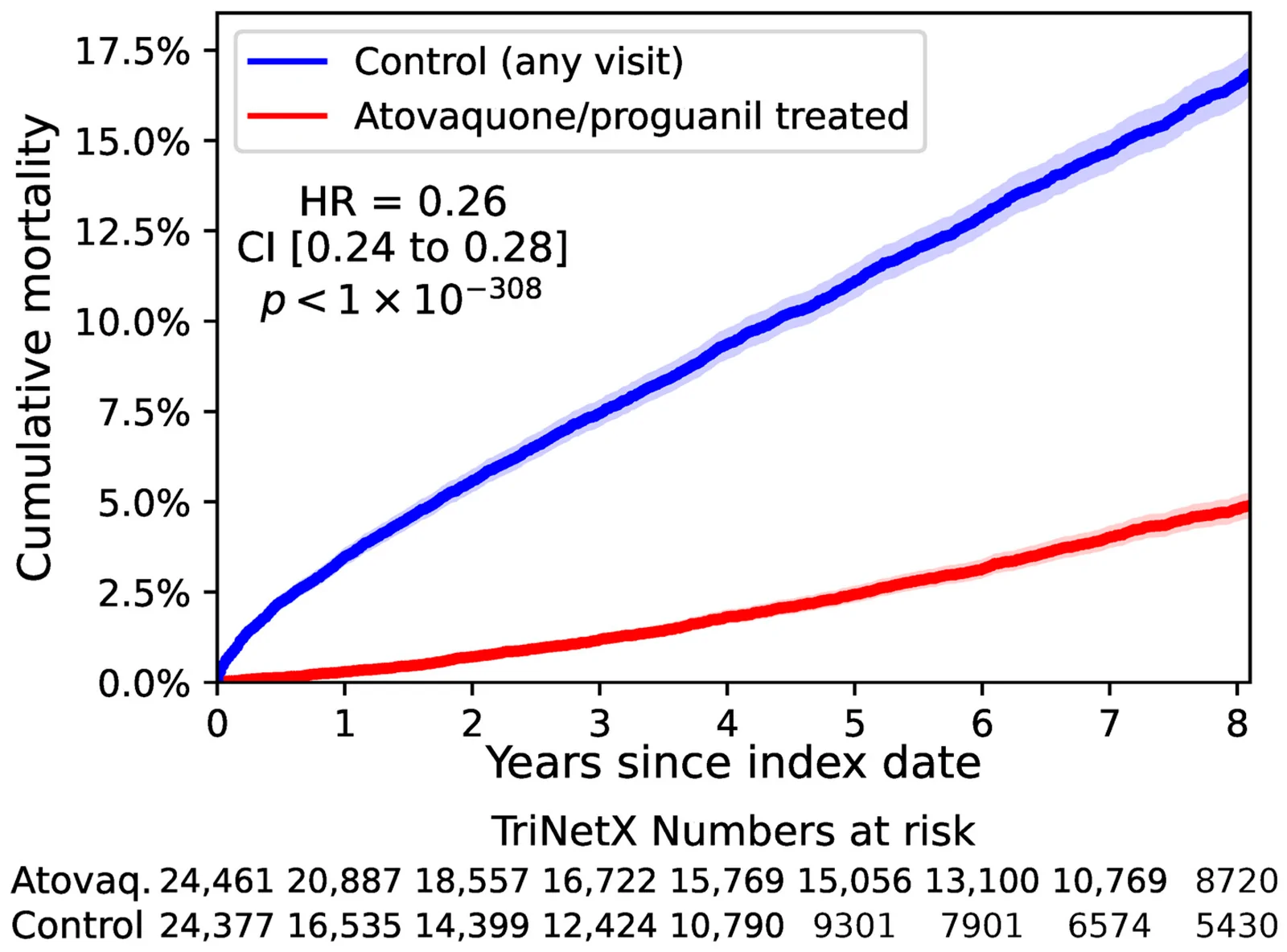
Sustained protozoal suppression is associated with reduced long-term human mortality. Cumulative all-cause mortality in propensity-score–matched cohorts exposed to atovaquone–proguanil (A-P) versus matched controls in the TriNetX real-world network (24,461 A-P users; 24,377 controls), followed for 8 years after the index exposure. Both arms were matched on age (69.0 ± 5.8), sex (55% female), race, ethnicity, smoking status, diabetes diagnosis, body mass index, and baseline HbA1c, and exhibited nearly identical distributions for each variable. Index dates were calendar-matched, eliminating bias from temporal effects such as seasonal mortality or pandemic timing. After matching, A-P exposure was associated with a 74% relative reduction in mortality (HR = 0.26; 95% CI 0.24–0.28; *p* < 1 × 10^−300^). This long-lasting effect far exceeds the drug’s short dosing duration and half-life, supporting the interpretation that the benefit arises not from temporary chemoprophylaxis but from suppression of a persistent biological risk factor, consistent with reduced pathogenic expression of latent protozoa such as *Toxoplasma gondii*.

In a national cohort study performed within Leumit Health Services (LHS) in Israel to identify drugs associated with increased lifespan, exposure to atovaquone–proguanil (A-P) or mefloquine, two widely used malarial prophylactic agents, was associated with large and sustained reductions in all-cause mortality over the subsequent decade [33] (Figure 1).

Both atovaquone and mefloquine inhibit a mitochondrial electron transport step in apicomplexan protozoa, including both *Plasmodium* species and *T. gondii*. Proguanil augments this effect by inhibiting dihydrofolate reductase and thereby potentiating mitochondrial collapse [34,35]. Beyond reduced all-cause mortality, protective associations were observed for diseases extending across dementia, metabolic disease, cardiovascular events, liver and renal failure, and colorectal and lung cancer. Statistical analyses employed rigorous pharmacoepidemiologic safeguards, including aligned index dates and adjustment for baseline comorbidity, with sensitivity analyses demonstrating close balance in baseline health-care utilization between exposed and comparator groups.

Each of these associations was independently replicated in the TriNetX federated network of approximately 120 million U.S. patients, using cohorts rigorously matched on age, sex, calendar time, and major clinical and demographic variables. Consistency across health systems and populations makes simple travel- or demographic-based confounding unlikely, particularly in light of strong decreases in mortality observed despite close balance of all known major factors of human morbidity at baseline.

These drugs were prescribed for their primary indication, malaria prophylaxis, but the multi-week courses typically used in this context provide a biologically plausible means of suppressing latent *Toxoplasma* burden in long-lived tissue reservoirs, with effects that may persist long after drug discontinuation. While residual confounding (including healthy traveler effects and unmeasured socioeconomic or behavioral factors) cannot be fully excluded, the parsimonious interpretation of the observed reductions in mortality and morbidity is that these outcomes share a previously underrecognized common substrate: a silent protozoal infection with delayed pathogenic effects, whose burden was reduced by these antiprotozoal treatments. The observed epidemiologic signals may therefore reflect attenuation of the parasite’s contribution to late-life impairment, a pattern consistent with the evolutionary constraints of *T. gondii*, whose reproductive success depends on its ability to facilitate felid predation or scavenging of the host at older ages.

If late-life morbidity is attributable to the parasite, pharmacologic suppression provides the most direct test available in humans. If reducing parasite burden reduces late-life disease, the late-life disease was in part parasite-driven. By suppression we mean pharmacologic reduction in tissue burden or of the frequency of reactivation, not necessarily eradication, as no available agent has been shown to completely eliminate bradyzoite cysts.

#### Mefloquine Exposure Is Associated with Reduced *T. gondii* Seropositivity in Young Adults

An additional clue linking antiprotozoal exposure to *T. gondii* biology in humans comes from a matched case–control analysis performed at LHS [36]. We compared individuals with a first documented positive *Toxoplasma* serology result (IgG or IgM) to individuals with consistently negative serology across all tests. We limited the analysis to patients aged 18 to 45 years without any diagnosis of immune deficiency, including HIV infection or use of immunosuppressive treatments, and without any recorded diagnosis of clinical toxoplasmosis at any time. After exclusions and exact 1:1 matching (on sex, age at test, year of first EHR documentation, socioeconomic status, ethnic group, and pregnancy status), without reuse of participants, the final analytical dataset included 19,443 seropositive individuals and 19,443 matched seronegative controls. The population was predominantly female (96.7%), with a mean age of 29.2 ± 6.1 years in both groups, reflecting primarily routine pregnancy screening aimed at detecting asymptomatic seroconversion during pregnancy to prevent potential congenital infection and fetal harm.

Exposure to mefloquine in the decade preceding serology testing was substantially less common among seropositive individuals than among matched seronegative controls (OR 0.45; 95% confidence interval (CI) 0.27–0.74, *p* = 0.002), consistent with an antiprotozoal effect of mefloquine on *T. gondii* persistence. In young adults (mostly pregnant women), *T. gondii* seropositivity is not associated with poorer health, and this further argues against a ‘healthy traveler’ explanation for the mefloquine protective association. Moreover, travel to malaria-endemic regions often entails conditions expected to increase, rather than decrease, exposure to food- and water-borne pathogens. The observed directionality therefore provides a simple epidemiologic clue that even time-limited exposure to antiprotozoals used for malaria prophylaxis years earlier can markedly suppress latent *T. gondii* persistence, in a setting where healthy traveler bias is unlikely.

### 4.2. Schizophrenia

Schizophrenia is an incompletely understood disease, associated with disordered thought processes and cognitive impairment, for which higher rates of *T. gondii* seropositivity have been detected across multiple populations [37,38,39,40], along with immune and metabolic abnormalities [41,42,43,44,45]. In addition to schizophrenia, *T. gondii* seropositivity has been associated with a broader spectrum of neuropsychiatric conditions, including suicidality, substance use disorders, obsessive–compulsive disorder, and bipolar disorder, suggesting a wider neurobehavioral footprint consistent with a common underlying mechanism [40,46,47,48]. If persistent toxoplasmosis contributes to schizophrenia risk, two predictions follow: suppression of the parasite should be protective, and impairment of host mechanisms that normally contain intracellular pathogens should increase risk. Both patterns were observed in an LHS cohort study [49].

The host mechanisms in question can be specified. Restriction of *T. gondii* is cell-autonomous and interferon-γ-dependent: host GTPases, together with ubiquitin-like conjugation systems of autophagy, load onto the parasitophorous vacuole membrane and disrupt it—immunity-related GTPases in murine cells, and GBP1 in human cells, which lack functional IRGs [50,51,52,53]. Above the cellular level, CD4+ and CD8+ T cells and interferon-γ maintain the cyst stage, and their loss produces reactivation encephalitis. That the vacuole is contested rather than sovereign is shown by the parasite itself, which encodes the kinase ROP18 and the pseudokinase ROP5 to keep these GTPases off the vacuolar membrane [23,24]: dedicated effector machinery does not evolve against a mechanism that poses no threat.

In a medication-wide screen covering all drugs dispensed in the decade before first schizophrenia diagnosis, the strongest protective associations clustered around agents with known anti-*Toxoplasma* activity, including atovaquone–proguanil used for malaria prophylaxis, clindamycin-based regimens used for acne, and moxifloxacin and oxytetracycline/polymyxin-B ophthalmic drops used to treat eye infections or for perioperative prophylaxis. Each of these associations was independently replicated in the TriNetX network with similar effect sizes and directionality. An independent study also reported a protective association for doxycycline, another tetracycline with anti-*Toxoplasma* effects [54]. The relevance of ophthalmic antibiotics, despite their limited systemic absorption, to pathogenic processes affecting the brain is discussed in detail in [49].

Host biological context further supports this interpretation. Before diagnosis, individuals who later developed schizophrenia showed lower vitamin D and T3 levels, higher rates of liver disease and alcoholism, reduced mucosal and barrier integrity, and increased prevalence of chronic viral hepatitis, whereas hepatitis A vaccination and episodes of transient mucosal inflammation were protective. Together, these features indicate impaired immune containment of intracellular parasites [43], a milieu in which latent *T. gondii* may shift from quiescence to clinically relevant neurobiological effects.

### 4.3. Heterogeneous, Stochastic Pathogenicity as an Adaptive Strategy

Under the delayed-pathogenicity framework, cognitive impairment and spatial disorientation represent phenotypes that likely increase vulnerability to predation or scavenging and thereby enhance felid consumption of infected hosts. However, stereotyped debilitation could destabilize predator–prey dynamics. *T. gondii* circulates across ecosystems containing multiple felid predators occupying distinct hunting and scavenging niches. If it were to produce a uniform pattern of host impairment that disproportionately benefits predators exploiting cognitively impaired prey over other felid predators or scavengers, this would restrict the ecological contexts in which *T. gondii*’s sexual reproduction can occur. Instead, a predation-dependent parasite would be expected to benefit from heterogeneity in the expression of host impairment, so that scattered disoriented individuals become available to predators exploiting this vulnerability, while other infected hosts follow different trajectories of decline aligned with alternative feline feeding strategies. Such diversification could provide a steady flow of prey across multiple feline ecological pathways, promoting sustained parasite sexual reproduction in diverse felid species.

A natural source of stochasticity that may provide diversity in host-impairment pathways and timing could be a viral infection reaching tissues in which *T. gondii* maintains a latent presence. At the population level, viral seasonality, epidemic waves, and host immune variability introduce heterogeneity in viral exposure across individuals. Within hosts, viruses spread locally, and some viruses, particularly herpesviruses, typically maintain latency in long-lived tissues and can reactivate focally as immune control weakens, introducing variation in tissue localization and intensity. Sporadic reactivation of varicella zoster virus (VZV) affecting a particular dorsal root ganglion produces shingles, a painful example of age-dependent focal viral reactivation. Analogous reactivation within the brain may occur silently. Such focal viral reactivations could provide the stochastic signal that modulates the timing and type of disability produced by *T. gondii* reactivation, while also serving as a biologic readout of immune weakening indicative of an aging host, for which predation has become advantageous. Conversely, the coinfection of tissues with oncogenic human papillomavirus strains may provide the stochastic signal that modulates *T. gondii*’s contribution to oncogenic pathways ultimately producing malignancy, as another means to debilitate the host at older age.

### 4.4. Dementia, Viral Triggers, and Delayed Protozoal Pathogenicity

*T. gondii* seropositivity has been reported to be higher across the dementia spectrum, from mild cognitive impairment to Alzheimer’s disease [55,56,57]. If latent toxoplasmosis contributes to late-life neurodegeneration, then interventions that suppress the parasite or prevent its reactivation should reduce dementia risk, whereas conditions that destabilize immune control should increase it. This pattern has been observed. In an unsupervised medication-wide screen performed in the U.K., a significantly lower incidence of Alzheimer’s disease was detected among individuals prescribed atovaquone–proguanil (A-P) or mefloquine, both CNS-penetrant antiprotozoals [58]. The protective association with A-P was independently detected in LHS and further replicated in large, age-stratified, rigorously matched U.S. TriNetX cohorts, where protection persisted for more than 10 years after exposure across multiple age strata [59].

Independent evidence points to a viral contributing effect. Varicella–zoster virus (VZV) vaccination is associated with reduced dementia risk [60,61], and herpesvirus genomic signatures are enriched in Alzheimer’s disease brain tissue, while antiviral therapy is associated with lower dementia incidence [62,63]. Experimental and clinical studies show that herpesvirus infection can impair immune control of latent *T. gondii* and induce protozoal reactivation within the central nervous system [64].

**Figure 2:**
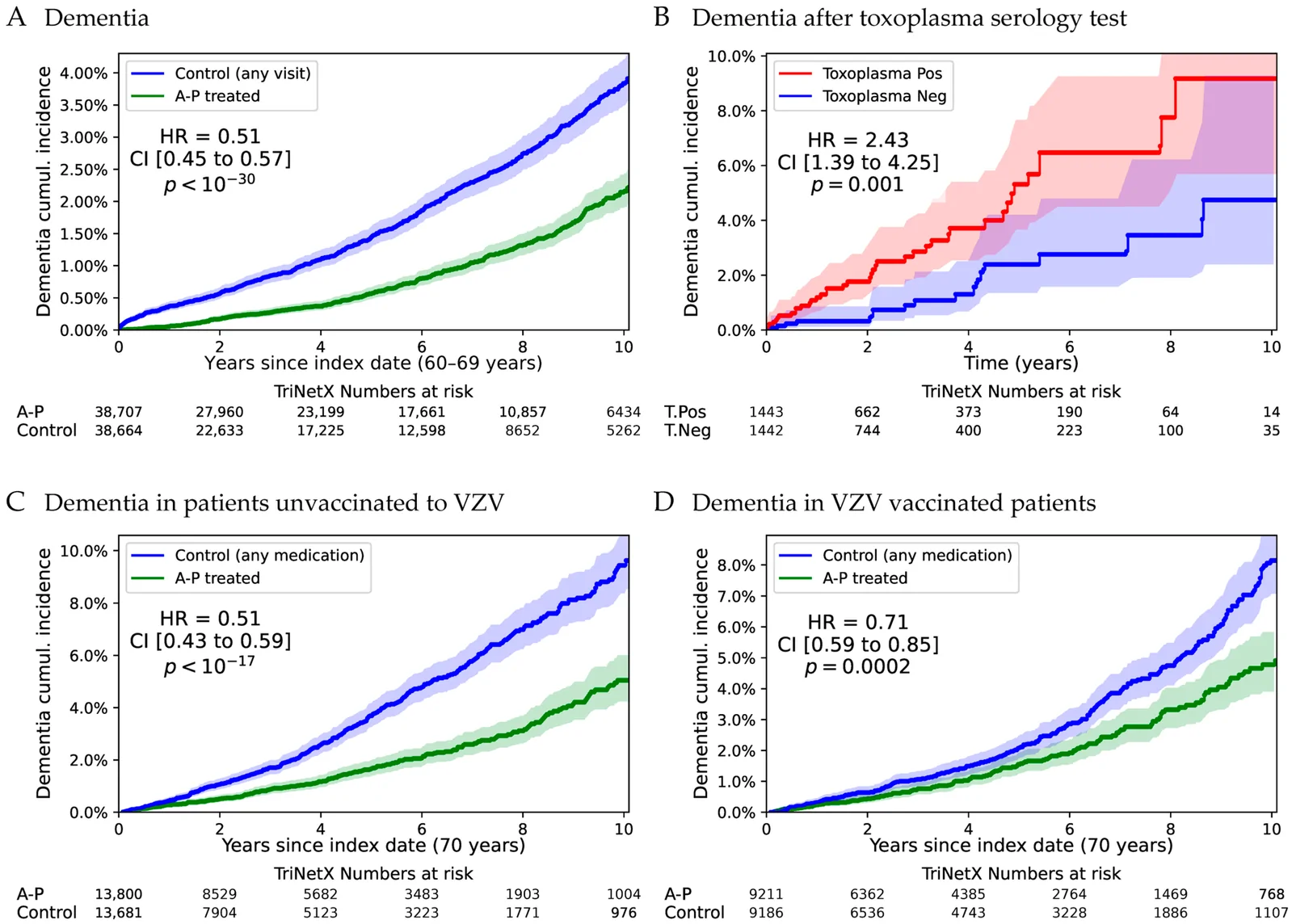
Viral activation of latent *Toxoplasma gondii* may facilitate neurodegeneration (**A**) Dementia incidence in propensity-score–matched cohorts exposed to atovaquone–proguanil (A-P) versus matched controls. A-P use was associated with significantly lower dementia risk after long-term follow-up (HR = 0.51; 95% CI 0.45–0.57, *p* < 10^−30^), despite the short preventive course and lack of any neurological indication. (**B**) Dementia incidence after *Toxoplasma gondii* serology testing. Individuals seropositive for *T. gondii* showed a significantly higher risk of future dementia (HR = 2.43; 95% CI 1.39–4.25, *p* = 0.001), indicating that infection precedes and is associated with the disorder rather than arising secondarily to prodromal decline. (**C**,**D**) Pathogen–pathogen interaction. The protective effect of A-P was significantly stronger in individuals not vaccinated against varicella-zoster virus (VZV) ((**C**); HR = 0.51; 95% CI 0.43–0.59, *p* < 10^−17^) than in those previously vaccinated ((**D**); HR = 0.71; 95% CI 0.59–0.85, *p* = 0.0002). Because VZV vaccination suppresses viral replication, these results are consistent with a model in which herpesvirus reactivation could facilitate protozoal transition from neuronal latency to pathogenic expression, providing a mechanistic route from lifelong infection to late-life neurodegeneration.

Consistent with this interaction model, the protective association of A-P was observed mostly for individuals who had not received VZV vaccination, and the protective association of VZV vaccination was observed mostly among those not exposed to A-P, suggesting that both act on the same downstream pathogenic pathway (Figure 2).

Longitudinal serologic data further support the framework: individuals seropositive for *T. gondii* had higher subsequent risk of dementia, with serology preceding diagnosis by many years. Together, medication, vaccination, and serologic evidence converge on a coherent model in which latent protozoal persistence, through episodic reactivation enabled by viral coinfection, contributes to stochastic late-life neurodegeneration.

### 4.5. Cancer

Increased *T. gondii* seropositivity has been observed in large prospective cohorts, notably in colorectal cancer and glioma, with timing indicating that infection occurs before tumor development [65,66,67]. Like neurodegeneration, malignancy risk increases with age and impairs host fitness. It would be expected to facilitate predation, by reducing the host’s ability to evade predators, and scavenging if the host dies of the disease. If latent toxoplasmosis contributes to carcinogenesis, then protozoal suppression should reduce cancer risk, and this pattern has been observed.

In Sweden, use of atovaquone–proguanil (A-P) was associated with reduced colorectal cancer incidence [68]. This protective association was independently replicated in the TriNetX network, where reduced risk of gastrointestinal malignancies persisted for up to 10 years after exposure and extended to pancreatic cancer [69]. The durability of protection following brief prophylaxis is more consistent with suppression of a persistent carcinogenic factor than with a direct tissue effect occurring only during drug exposure.

**Figure 3:**
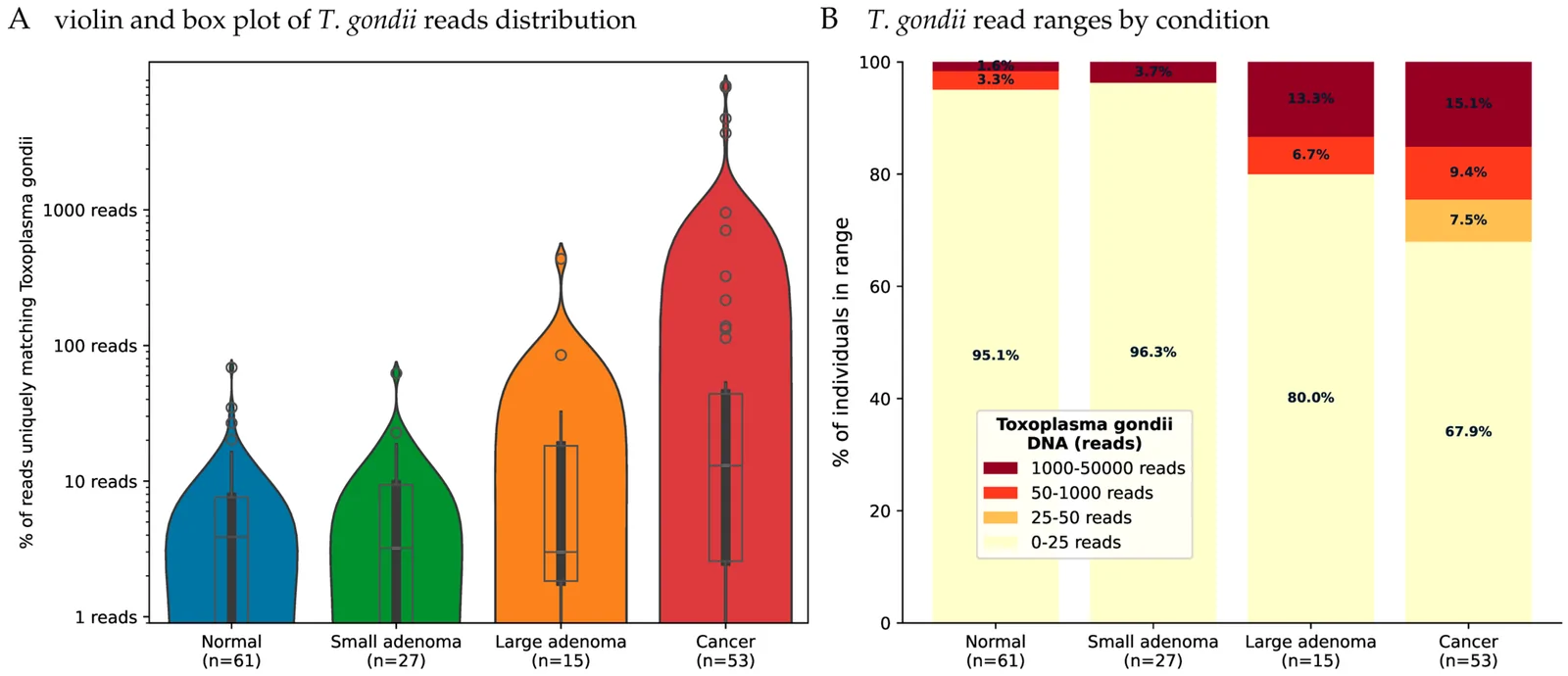
Progressive enrichment of *Toxoplasma gondii* DNA across colorectal neoplasia. (**A**) Violin and box plots displaying uniquely mapped *T. gondii* reads at ≥1-read threshold across normal mucosa (n = 13), small adenomas (n = 127), large adenomas (n = 127), and colorectal cancer (n = 143). Abundance increases stepwise from normal tissue to adenoma to carcinoma. (**B**) Distribution of individuals by read-range category shows an escalating proportion with high read burden (≥50 to >1000 reads) in advanced lesions, rising from 0% in controls to 15.1% in small adenomas, 33.1% in large adenomas, and 47.9% in colorectal cancers. Together, the gradient is consistent with progressive tissue-level accumulation before malignant transformation, compatible with a role in tumor initiation or early progression. Reproduced from Israel et al., Gut Microbes (2025) [69], under the terms of the Creative Commons Attribution-NonCommercial License (CC BY-NC 4.0).

Microbiome sequencing experiments provide complementary clues. Reanalysis of a large colorectal cancer metagenomic dataset (PRJEB6070) using protozoa-inclusive reference libraries identified *T. gondii* as the single most discriminatory taxon, exceeding *Fusobacterium*, which was the most discriminatory taxon in the original study [69]. *T. gondii* DNA was detected in 22.1% of cancer samples compared with 1.53% of controls, with abundance increasing progressively from normal mucosa to adenoma to carcinoma (Figure 3).

Together, prospective serology, durable pharmacoepidemiologic protection, and direct detection of parasite DNA along the adenoma–carcinoma sequence converge on a coherent model in which persistent toxoplasmosis is associated with malignant transformation in the human gut and possibly beyond.

## 5. Testable Predictions and Research Roadmap

If latent *T. gondii* contributes meaningfully to age-associated morbidity, with pathogenic expression delayed until later life, then several empirically verifiable and potentially falsifiable predictions emerge.

### 5.1. Molecular Enrichment in Disease-Relevant Tissues

#### 5.1.1. Tissue Colonization Would Be Associated with Disease

Diseases suspected of being influenced by latent *Toxoplasma* would be expected to demonstrate higher rates of detectable parasite DNA or RNA in relevant tissues, particularly along progressive biological gradients (premalignant lesions, preclinical neurodegeneration, subclinical vascular injury). Importantly, colonization may be compartmentalized, meaning that absence of serologic response does not exclude tissue-level persistence. Demonstrating tissue-embedded parasites prior to clinical onset would provide strong evidence against incidental contamination.

#### 5.1.2. Tissue-Specific Latent Infection May Be Detected Non-Invasively

If latency represents an active biological program rather than inert dormancy, parasite-specific metabolic pathways may be detectable using immunohistochemical markers or PET tracers targeting apicomplexan mitochondria, cyst-wall glycans, or *Toxoplasma*-specific enzymes. Demonstrating spatial colocalization between tracer uptake and disease-relevant pathology would provide an independent validation layer beyond molecular detection alone.

### 5.2. Interventional Proof Through Controlled Suppression

#### 5.2.1. Short-Course Suppressive Regimens May Yield Durable Benefit

Randomized trials in individuals with evidence of latent infection may test whether suppressive anti-*Toxoplasma* regimens produce durable reductions in selected aging-related outcomes, including dementia, malignancy, cardiovascular events, metabolic disease, or mortality. If benefits persist beyond the treatment period and correlate with reduced molecular signatures of parasite activity, this would provide compelling evidence consistent with causality. Conversely, the absence of a measurable durable effect would challenge the delayed-pathogenicity framework.

#### 5.2.2. Pathogenic Transition Is Expected to Be Regulated by Hormonal Changes Associated with the End of Reproductive Phase

Since natural selection would be expected to favor host impairment that is delayed until after active reproduction, if delayed pathogenicity is adaptive, *T. gondii* developmental transitions may be influenced by endocrine signals associated with reproductive status and aging. Transition to pathogenicity may be inhibited by hormones associated with reproductive activity and enabled by menopause and other endocrine changes accompanying aging. Candidate signals include alterations in the GnRH axis, sex steroid trajectories (progesterone, estrogens, testosterone, DHEA)—which have already been shown to directly influence *T. gondii* motility and egress [70]—as well as pregnancy-associated hormones (hCG, hPL, relaxin) and age-associated changes in thyroid and growth-hormone signaling. This hypothesis can be evaluated in animal models and mechanistic studies, and in human cohorts by examining endocrine trajectories preceding outcomes plausibly linked to *T. gondii*-mediated impairment.

### 5.3. Reconsidering Serology as a Marker of Latent Infection

Serologic positivity is commonly treated as the canonical marker of *Toxoplasma* infection status. However, this assumption has important limitations. Immune responses vary substantially across hosts, and persistent infection confined to localized tissue niches may, in some circumstances, fail to elicit or maintain a durable serologic response. Moreover, seropositivity may behave as a continuum rather than a binary state. Strong antibody responses may reflect ongoing immune engagement, whereas lower antibody titers, potentially below standard positivity thresholds, could in some individuals reflect reduced immune surveillance rather than complete absence of persistent infection.

Under the delayed-pathogenicity framework proposed here, long-term persistence within localized host niches could provide a means of minimizing immune detection while maintaining the potential for later pathogenic expression. Confirming this hypothesis would require direct validation through tissue-based molecular studies.

Accordingly, future investigations should integrate multiple markers of persistence, including tissue-level molecular detection, immune signatures, and imaging biomarkers. Serology remains informative, but it likely captures only part of the biological spectrum of persistent *T. gondii* infection.

### 5.4. Ecological and Life-History Measurements in Natural Intermediate Hosts

The evolutionary claim cannot be tested in humans. It requires, in prey species subject to felid predation: age-stratified prevalence and incidence; age-specific reproductive value; predation and scavenging rates as a function of infection status and host age; and the age, or the interval after infection, at which transmission-relevant impairment first appears. If impairment appears at ages of high reproductive value, the framework is wrong. These are demanding but conventional field-ecological measurements, and to our knowledge they have not been assembled for any single host population.

### 5.5. Experiments Discriminating Delayed Pathogenicity from Immunosenescence

Two experiments separate the framework advanced here from the null model of Section 6. In the first, young reproductively active intermediate hosts are immunosuppressed to a defined degree and compared to post-reproductive hosts matched for the degree of immune impairment. The null model predicts that the full late-life phenotype is reproduced in the young animals; the delayed-pathogenicity model predicts attenuated or delayed pathogenic transition relative to the older animals.

In the second, and more informative, experiment, young immunocompetent hosts undergo gonadectomy or pharmacologic suppression of reproductive hormones. The delayed-pathogenicity model predicts acceleration of the bradyzoite-to-tachyzoite transition and earlier appearance of tissue pathology; the null model predicts no effect, since immune competence is unchanged. Existing evidence that estradiol and progesterone directly influence *T. gondii* motility, microneme secretion, and egress [70] makes such an effect biologically plausible without presupposing it. We regard this as the single most informative test of the present hypothesis. A null result would falsify the claim that the timing of pathogenic expression is under parasite-encoded, reproduction-linked control, and would leave immunosenescence as the sufficient explanation.

## 6. Alternative Interpretations and Limitations

The framework should be distinguished from its alternatives. The relevant alternative is not that *T. gondii* is inert in humans; the observations of Section 4 are difficult to reconcile with that view, and arguing against it is the purpose of this paper. It is that late-life expression reflects the decay of interferon-γ-dependent restriction with age rather than any parasite program. Under this null model, the parasite schedules nothing, the timing is host-determined, and the human data of Section 4 follow equally well. Two considerations bound it. It is silent on the observation that motivates the hypothesis—why a parasite whose most productive transmission route entails the death of an intermediate host produces so little acute mortality across so broad a host range—and it does not predict the direct effects of estradiol and progesterone on *T. gondii* motility, microneme secretion, and egress [70]. The two models diverge in one testable respect: the null makes timing a function of immune status alone, whereas the framework advanced here predicts residual dependence on host reproductive status. Section 5.5 exploits that divergence.

The agents whose protective associations we report are not clean pharmacologic probes of parasite biology. Atovaquone inhibits gp130-dependent STAT3 phosphorylation, alters myeloid-derived suppressor and regulatory T-cell populations, and binds mammalian cytochrome bc1, though with far lower affinity than the apicomplexan enzyme [71,72,73]. An immunomodulatory or metabolic mechanism unrelated to parasite clearance is therefore not excluded. Four considerations constrain that explanation without refuting it: the protective associations were observed over a period far exceeding the duration of prophylactic treatment, which an immunomodulatory effect, being transient, does not readily explain; they span agents whose only shared property is activity against apicomplexan parasites; the mefloquine association was observed against a parasitologic endpoint in young adults, in whom healthy-traveler bias is implausible; and the enrichment of parasite DNA along the adenoma–carcinoma sequence is drug-independent. The trial of Section 5.2.1 remains the decisive test.

Two limitations remain. The framework does not apply uniformly across the host range, and we have not attempted to specify the species in which the necessary life-history conditions hold. And the human associations of Section 4 are observational: all measured confounders were balanced by matching and the associations replicated across independent health systems, but unmeasured confounding cannot be excluded on logical grounds alone—which is why an interventional test, rather than any further observational analysis, is required to establish causation.

## 7. Conclusions: A Parasite Optimized for Delayed Morbidity

Across independent disease domains, convergent evidence summarized in Table 1 reveals a coherent and internally consistent pattern suggesting *T. gondii* involvement in a wide range of pathogenetic processes. Few existing host-centered models readily account for the breadth of this cross-domain convergence. In contrast, parasite-mediated health deterioration emerging later in life is broadly consistent with the evolutionary constraints imposed by the *T. gondii* life cycle. Because the parasite can complete sexual reproduction only when infected host tissue is consumed by a felid, selective pressures may favor traits that promote health or behavioral impairment predominantly in later life, when predation or scavenging becomes advantageous for parasite transmission without substantially compromising host population persistence.

Humans are frequently and repeatedly exposed to *T. gondii* through environmental oocysts in soil, water, and fresh produce, as well as through consumption of undercooked meat, unpasteurized milk [74] and shellfish [75]. As incidental intermediate hosts, humans may experience deleterious pathogenic effects selected by evolution in other warm-blooded species to facilitate felid consumption of infected hosts. Accordingly, *T. gondii*-induced pathogenicity may manifest as health disorders of later life, including vascular, metabolic, neurodegenerative, and malignant disease. Long-term tissue persistence with minimal replication and immune activation, delayed pathogenicity, and the possibility that serologic responses may fail to capture low-grade or tissue-restricted infection raise the possibility that a major contributor to human morbidity could have remained largely unrecognized.

Together, evolutionary logic and human data support a testable hypothesis: a measurable fraction of late-life morbidity may reflect delayed consequences of *T. gondii* infection rather than purely intrinsic processes. If correct, at least some age-related diseases may depend on targetable infectious factors [76], with substantial health implications. Suppressing latent protozoal infection, preventing reactivation, or interrupting transmission could provide a practical means of promoting healthy aging by targeting an upstream ecological driver of multisystem morbidity. Importantly, this hypothesis yields specific falsifiable predictions that can be evaluated using controlled cohort studies and metagenomic, molecular, and metabolic analyses.

A closing word on the status of this proposal. This is a hypothesis article, and what is advanced here is a conjecture rather than a demonstrated result. The human data reviewed are associational and cannot by themselves establish causation; Section 6 states the principal alternative interpretation, and Section 5.5 specifies the experiment whose failure would falsify the framework. A hypothesis should be judged on its internal coherence, its consistency with established parasitology, and its falsifiability.

## Figures and Tables

**Table 1:** Convergent evidence supporting delayed pathogenicity of *Toxoplasma gondii.* Summary of epidemiologic, molecular, and evolutionary observations and their interpretation under the delayed-pathogenicity hypothesis; inclusion does not imply proof of causality.

Domain	Observation	Interpretation
**Parasite ecology**	Sexual reproduction occurs only in felids following ingestion of infected tissue; this route also yields the only environmentally durable stage—oocysts shed in the hundreds of millions and infective for 18 months in soil and 24 months in seawater—whereas the alternative routes of felid infection yield no comparable amplification [3,5,6,7,8]	*T. gondii* reproductive success is contingent on host death. The route requiring host death is also the one that disperses the parasite most widely and for longest.
**Behavioral alteration**	Infected rodents lose their aversion to cat odor and can be attracted to it [16,17]	*T. gondii* expresses traits that facilitate felid-mediated predation and host death; rodent-specific behavioral manipulation does not explain transmission through other host species
**Evolutionary logic**	Host impairment occurring in early life would disrupt host reproduction and collapse transmission reservoirs [10,11,21,22]	Pathogenic effects would be predicted to emerge preferentially after attainment of adult size and completion of the reproductive phase required for population persistence
**Latency biology**	Long-lived, immune-quiet bradyzoite cysts escape immune clearance and persist in neural and muscular tissue [30,31]	Consistent with an evolutionary adaptation enabling persistence until stages of host life at which predation may become advantageous for transmission
**Human mortality and morbidity**	Pharmacologic protozoal suppression is associated with large, sustained reductions in all-cause mortality and multiple chronic diseases [33].	An epidemiologic signal consistent with a substantial contribution of protozoa to human late-life morbidity and mortality
**Schizophrenia**	*T. gondii* seropositivity is increased in schizophrenia; anti-*Toxoplasma* drugs are associated with decreased schizophrenia occurrence; immune and barrier deficits are associated with increased risk [37,38,39,40,41,42,43,44,45,49]	Neuropsychiatric impairment appears to emerge when central nervous system containment of *T. gondii* is compromised, a host state that precedes disease onset
**Dementia**	Atovaquone–proguanil, mefloquine and VZV vaccination are protective; *T. gondii* seropositivity precedes disease [55,56,57,58,59,60,61]	Age-associated cognitive decline is linked to the combined presence of protozoal persistence and uncontained neurotropic viral infection
**Cancer**	Protozoal suppression is protective, seropositivity precedes diagnosis, and *T. gondii* DNA increases along the adenoma–carcinoma sequence [65,66,67,68,69].	This pattern is consistent with a role of *T. gondii* in the malignant transformation process

## Data Availability

Data supporting the referenced publications are available as described in those articles and their associated public repositories. Individual-level patient data from Leumit Health Services are not publicly available due to privacy and regulatory restrictions, but may be made available upon reasonable request to qualified researchers, subject to IRB approval.

## Abbreviations

A-P	Atovaquone–Proguanil
BMI	Body mass Index
CI	Confidence Interval
CRC	colorectal cancer
DM	Diabetes Mellitus
EHRs	electronic health records
HR	Hazard ratios
LHS	Leumit Health Services
T3	triiodothyronine
VZV	Varicella Zoster Virus
